# The Potential Role of CA-125 as a Biomarker for Short-Term Mortality Risk in Patients with Acute Symptomatic Pulmonary Embolism

**DOI:** 10.3390/jcm13123601

**Published:** 2024-06-20

**Authors:** Crhistian-Mario Oblitas, Francisco Galeano-Valle, Marta-Olimpia Lago-Rodríguez, Marina López-Rubio, Jesús Baltasar-Corral, Mercedes García-Gámiz, Angielys Zamora-Trillo, Luis-Antonio Alvarez-Sala Walther, Pablo Demelo-Rodríguez

**Affiliations:** 1 Venous Thromboembolism Unit, Internal Medicine Department, General University Hospital Gregorio Marañón, 28007 Madrid, Spain; crhistian.cao@gmail.com (C.-M.O.); mlagorodr@gmail.com (M.-O.L.-R.); marinalopezrubio@outlook.com (M.L.-R.); jesusbaltasarcorral@gmail.com (J.B.-C.); lalvarezsalaw@gmail.com (L.-A.A.-S.W.); pbdemelo@hotmail.com (P.D.-R.); 2 School of Medicine, University Complutense of Madrid, 28007 Madrid, Spain; 3 Instituto de Investigación Sanitaria Gregorio Marañón, 28007 Madrid, Spain; 4 Department of Clinical Biochemistry, General University Hospital Gregorio Marañón, 28007 Madrid, Spain; mggamiz@salud.madrid.org (M.G.-G.); dra.angie62@gmail.com (A.Z.-T.)

**Keywords:** bleeding, CA-125, mortality, pulmonary embolism, venous thromboembolism

## Abstract

**Background**: Antigen carbohydrate 125 (CA-125) is a complex glycoprotein extensively studied as a prognostic biomarker in heart failure, yet its potential role in the short-term prognosis of an acute pulmonary embolism (PE) remains unexplored. **Methods**: In this observational, prospective, single-center study, consecutive patients aged 18 and older with a confirmed acute symptomatic PE and no history of prior anticoagulant therapy were enrolled. Primary and secondary objectives aimed to assess the prognostic capacity of CA-125 at PE diagnosis for 30-day mortality and major bleeding, respectively. **Results**: A total of 164 patients were included (mean age 69.8 years, SD 17), with 56.1% being male. Within 30 days, 17 patients (10.4%) died and 9 patients (5.5%) suffered major bleeding. ROC curve analysis for 30-day mortality yielded an area under the curve of 0.69 (95% CI 0.53–0.85) with an optimal CA-125 cut-off point of 20 U/mL and a negative predictive value of 96%. Multivariate analysis revealed a significant association between CA-125 levels exceeding 20 U/mL and 30-day mortality (adjusted odds ratio 4.95; 95% CI 1.61–15.2) after adjusting for age, cancer, NT-proBNP > 600 ng/mL, and the simplified pulmonary embolism severity index score. Survival analysis for 30-day mortality exhibited a hazard ratio of 5.47 (95% CI 1.78–16.8). No association between CA-125 levels and 30-day major bleeding was found. **Conclusions**: CA-125 emerges as a promising surrogate biomarker for short-term mortality prediction in an acute symptomatic PE. Future investigations should explore the integration of CA-125 into PE mortality prediction scores to enhance the prognostic accuracy in this patient population.

## 1. Introduction

A venous thromboembolism (VTE) represents a chronic condition associated with a potentially significant burden of mortality and morbidity, ranking as the third cause of vascular diseases, following ischemic heart disease and cerebrovascular disease. This high burden is mainly attributed to a pulmonary embolism (PE) [1,2]. Over the past two decades, the incidence of PEs has doubled, increasing from 62 to 120 cases per 100,000 persons/year. This rise can be attributed to improved diagnostic techniques capable of detecting thrombosis in small vessels, as well as the identification of low-risk and incidental PE cases. Furthermore, age plays a substantial role in PE incidence, with individuals aged over 75 facing a sevenfold higher risk compared to those under 50 (450 vs. 50 cases per 100,000 persons/year) [1,2,3,4,5].

The development of VTE results from a complex interplay of environmental factors such as infections, cancer, and medical devices, among others, and intrinsic risk, whether hereditary or acquired. This intricate process involves the key components of Virchow’s triad, encompassing venous stasis, endothelial damage, and hypercoagulable states. Despite significant progress in our understanding of VTE, unraveling the underlying molecular mechanisms remains an ongoing challenge [1,2,4,6].

After the diagnosis of PE, assessing prognosis stratification in patients becomes essential, with particular attention to evaluating the hemodynamic state. Hemodynamically unstable or high-risk PE is defined as the presentation in the form of cardiorespiratory arrest, persistent arterial hypotension (systolic blood pressure [SBP] < 90 mmHg or a drop in SBP ≥ 40 mmHg for a period > 15 min in the absence of de novo arrhythmias, hypovolemia, or sepsis), or the presence of obstructive shock. This clinical presentation carries the highest risk for short-term mortality, and the treatment of choice is pharmacological fibrinolysis unless contraindicated. On the other hand, hemodynamically stable PEs with right-heart dysfunction (identified through CT angiography or an echocardiogram) and/or elevated cardiac biomarkers (high-sensitivity troponin, NT-proBNP) are categorized as intermediate-risk PEs according to the European Society of Cardiology (ESC) 2019 score. However, these patients constitute a heterogeneous group with varying prognoses in real-life scenarios. Therefore, there is a pressing need to explore new biomarkers for improved and precise risk stratification in clinical practice [2,3,4,5,7,8,9].

Antigen carbohydrate 125 (CA125), also known as cancer antigen 125 or the mucin 16 protein, has been widely utilized as a prognostic biomarker for ovarian cancer, but it is also elevated in other malignant entities (breast cancer, mesotheliomas, non-Hodgkin lymphomas, or leiomyosarcomas) as well as benign conditions such as heart failure (HF), chronic liver disease, tuberculosis, among others. Additionally, it appears to have an immunomodulatory role in different immune cells, primarily natural-killer lymphocytes, potentially contributing to the immunological tolerance of the mother during fetal implantation [10,11]. CA125 consists of three domains: a heavily glycosylated large N-terminal domain, a tandem repeat domain (serine, threonine, and proline) interspersed with the sea urchin sperm protein, the enterokinase and agrin (SEA) domain, and a C-terminal domain. CA125 is synthesized in mesothelial cells of the peritoneum, pleura, and pericardium in response to various stimuli, including inflammation, congestion states, and water overload, among others [9,10,12,13]. CA 125 exhibits a prolonged half-life, measured in weeks, and does not appear to be significantly influenced by age or kidney function. While its exact pathophysiology remains incompletely understood, recent research suggests a potential role as a prognostic biomarker in acute or chronic HF. This association is linked to hemodynamic changes (alterations in the cardiac cavity volume or pressure) and the inflammatory state proposed in this condition, which correlates with the presence of proinflammatory cytokines (IL-1, TNF alpha, IL-6, among others). Moreover, these changes might be detectable even in the absence of clinically evident serous effusion, offering the possibility of dynamic monitoring. Higher CA 125 levels tend to correspond with worsened clinical conditions, while the normalization of levels often follows clinical improvement or a favorable response to treatment [11,12,13,14].

This study aims to investigate whether elevated serum CA-125 levels at the time of acute symptomatic PE diagnosis can predict short-term mortality or, conversely, are associated with serious complications related to anticoagulant treatment within the first 30 days.

## 2. Materials and Methods

### 2.1. Study Design

This is an observational, prospective, single-center study that was conducted from April 2021 to September 2022 in a third-level hospital. We enrolled consecutive patients with a confirmed diagnosis of acute symptomatic pulmonary embolism by objective imaging tests (helicoidal computed tomography or scintigraphy).

Inclusion criteria were age 18 or older, confirmed PE with or without deep vein thrombosis by an objective imaging test, and signed informed consent. Exclusion criteria were incidental or asymptomatic pulmonary embolism, venous thromboembolism without pulmonary embolism, and patients on chronic anticoagulant therapy (at any dose or cause).

All patients were followed up for at least the first 30 days after PE diagnosis. Demographic characteristics, laboratory variables, and clinical outcomes were collected from electronic medical history.

### 2.2. Outcomes

The primary outcome was the capacity of CA-125 to predict all-cause mortality at short-term prognosis within the first 30 days, while the secondary outcome assessed its predictive value for 30-day major bleeding events. Major bleeding was defined as requiring transfusion of 2 or more units of blood, occurring at a critical site or contributing to death [15].

### 2.3. Sample Collection and Handling

Within the first 24 h after PE diagnosis, whole blood samples were collected in lithium heparin serum and plasma tubes from patients, centrifuged immediately after collection (1700× *g* at 4 °C for 15 min), and stored in two aliquots at −20 °C. Before analysis, samples were thawed and vortexed at low speed to ensure uniformity. CA-125 levels were determined using a two-step chemiluminescent microparticle immunoassay (CMIA) on the Abbott Alinity I analyzer. The measurement range of this parameter in our laboratory is 0–35 U/mL, with linearity between 1.1 U/mL and 1000.0 U/mL. Plasma CA-125 levels were measured once at PE diagnosis.

### 2.4. Statistical Analysis

Categorial data were reported as proportions and continuous data as means with standard deviation (SD) or median and the 25th (P25) and 75th (P75) percentiles or Interquartile range (IQR), depending on normality. Student *T*-test and analysis of variance (ANOVA) were used for normally distributed variables. Mann–Whitney U and Kruskal–Wallis tests were used for non-normally distributed variables. The Spearman rank-order correlation coefficient was calculated to assess the correlation between CA-125 and other biomarkers. The Kaplan–Meier estimator was performed for event visualization. Receiver operating characteristic (ROC) curve analysis determined the predictive capacity of CA-125, with Youden determining the optimal cut-off. Bivariate logistic regression calculated crude odds ratios (OR), while multivariate logistic regression assessed independent association with variables significant in the bivariate analysis (adjusted OR). All tests were two-sided and the level of statistical significance was set at 0.05. Statistical analysis was carried out using STATA software (v14.2).

### 2.5. Ethical Considerations

This study received approval from the local Ethics Committee. Informed consent was obtained from all enrolled patients, providing comprehensive information regarding the study’s purpose and potential risks. Patient data were anonymized to protect their privacy.

## 3. Results

The study enrolled a total of 164 patients, with a mean age of 69.8 years (SD 17), of whom 56.1% were male. Eighty-three patients (50.6%) presented arterial hypertension, 55 patients (33.5%) presented dyslipidemia, 32 patients (19.5%) presented chronic obstructive pulmonary disease, 22 patients (13.4%) had diabetes mellitus, and 14 patients (8.5%) had chronic heart failure. The laboratory findings showed a mean D-dimer level of 2648 ng/mL (IQR 1342–6000 ng/mL), mean NT-proBNP levels of 631 ng/L (IQR 212–3038 ng/L), and elevated levels of troponin-hs in 75 patients (45.7%). The baseline clinical and laboratory characteristics are summarized in Table 1.

Related to the risk stratification for a pulmonary embolism, high-risk PE was observed in eight patients (4.9%), while 54 patients (32.9%) had intermediate–high-risk PE, 64 patients (39%) had intermediate–low-risk PE, and 38 patients (23.2%) had low-risk PE, according to the ESC score. Sixty-nine patients (42.1%) exhibited the involvement of the trunk of the pulmonary artery or its main branches. Among the entire sample, 77 patients (46.9%) had no identifiable provoking factors, while 33 patients (20.1%) had associated cancer (five lung, five gastrointestinal, four hematological, four hepatobiliary, three renal, three ovarian, three breast, two brain, and five other tumors). Overall mortality occurred in 17 patients (10.4%) within 30 days and 9 patients (5.5%) suffered major bleeding. Non-survivors presented higher CA-125 levels (15.0 U/mL [IQR 10.3–27.7 U/mL] vs. 35.4 U/mL [IQR 20.2–108.9 U/mL]; *p* = 0.01).

**Figure 1:**
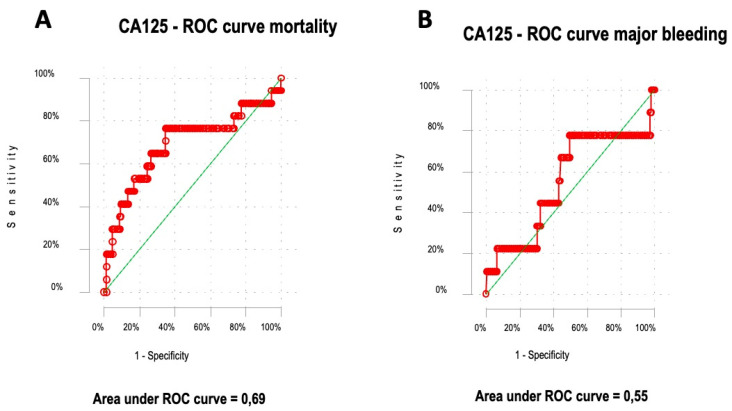
Predictive capacity of CA-125 for 30-day mortality (**A**). Predictive capacity of CA-125 for 30-day major bleeding (**B**). The red line in each graph represents the ROC (Receiver Operating Characteristic) curve for the CA125 marker in relation to the outcomes of mortality (Panel **A**) and major bleeding (Panel **B**). The green diagonal line represents the line of no-discrimination, which corresponds to an AUC (Area Under the Curve) of 0.5.

For the prediction of 30-day mortality, the ROC curve yielded an area under the curve (AUC) of 0.69 (95% confidence interval [CI] 0.53–0.85). The Youden index showed an optimal CA-125 cut-off point of 20 U/mL, with a negative predictive value (NPV) of 96% (Figure 1A). Conversely, the ROC curve for 30-day major bleeding produced an AUC of 0.55 (95% CI 0.33–0.78; *p* = 0.5) (Figure 1B). Additionally, there were no differences for CA-125 levels between those with or without right-ventricle dysfunction (16.8 U/mL [IQR 11.9–32.7 U/mL] vs. 13.6 U/mL [IQR 8.7–27.5 U/mL]; *p* = 0.17).

The Spearman rank-order correlation coefficient for CA-125 and others biomarkers showed no significant correlation between CA-125 and others biomarkers: CA-125 and NT-proBNP (Spearman’s rho 0.12; *p* = 0.17), CA-125 and CRP (Spearman’s rho 0.07; *p* = 0.36), CA-125 and D-dimer (Spearman’s rho −0.04; *p* = 0.59), or CA-125 and hemoglobin (Spearman’s rho −0.14; *p* = 0.08).

In the bivariate analysis, CA-125 levels greater than 20 U/mL demonstrated a statistically significant prognosis capacity for 30-day mortality (crude OR 6.1; 95% CI 1.89–19.8), while no significant association was observed in the bivariate analysis for 30-day major bleeding (crude OR 1.27; 95% CI 0.33–4.93) (Table 2).

**Figure 2:**
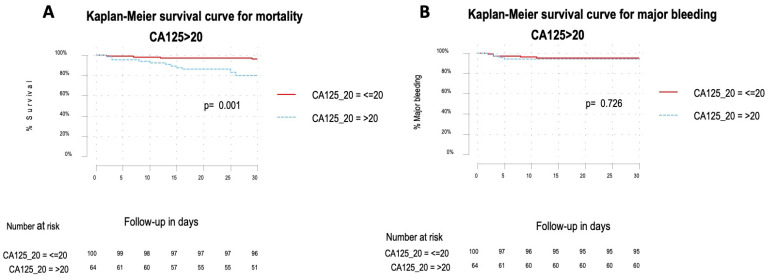
Overall survival Kaplan–Meier analyses of 30-day mortality for CA-125 > 20 U/mL showed a hazard ratio of 5.47 (95% CI 1.78–16.8). The absolute numbers of surviving patients on days 0, 5, 10, 15, 20, 25, and 30 comparing levels from above or below the optimal cut-off (**A**). Overall survival Kaplan–Meier analyses of 30-day major bleeding for CA-125 > 20 U/mL showed a hazard ratio of 1.26 (95% CI 0.34–4.68). The absolute numbers of surviving patients on days 0, 5, 10, 15, 20, 25, and 30 comparing levels from above or below the optimal cut-off (**B**).

In the multivariate analysis, CA-125 levels greater than 20 U/mL remained significantly associated with 30-day mortality (adjusted OR 4.95; 95% CI 1.61–15.2), after adjusting for age, cancer, NT-proBNP > 600 ng/mL, and the simplified pulmonary embolism severity index score (PESIs) (Table 3). The survival analysis for 30-day mortality demonstrated a hazard ratio (HR) of 5.47 (95% CI 1.78–16.8; *p* < 0.01) (Figure 2A), while no significant association was found for 30-day major bleeding (HR 1.26; 95% CI 0.34–4.68; *p* = 0.73) (Figure 2B).

## 4. Discussion

CA-125 exhibits a prolonged half-life, measured in weeks, and does not appear to be significantly influenced by age or kidney function. While its exact pathophysiology remains incompletely understood, recent research suggests a potential role as a prognostic biomarker in acute or chronic HF. This association is linked to hemodynamic changes (alterations in the cardiac cavity volume or pressure) and the inflammatory state proposed in this condition, which correlates with the presence of proinflammatory cytokines (IL-1, TNF alpha, IL-6, among others). Moreover, these changes might be detectable even in the absence of clinically evident serous effusion, offering the possibility of dynamic monitoring. Higher CA-125 levels tend to correspond with worsened clinical conditions, while the normalization of levels often follows clinical improvements or a favorable response to treatment [12,13,14]. The present study is the first to show that elevated CA-125 levels in acute symptomatic PE patients are associated with a fivefold higher risk of death within the first 30 days after diagnosis. Over the past two decades, CA-125 has garnered interest as a prognostic biomarker in various non-tumor pathologies, particularly in cardiorespiratory conditions such as HF and chronic obstructive pulmonary disease (COPD) [12,14]. However, its potential role in PE has remained unexplored until now. Studies in HF have yielded intriguing findings. Nägele et al. [16] investigated 71 patients with HF before and after heart transplantation (HTx), revealing a significant decrease in CA-125 levels post-HTx or during HF stabilization, whereas these levels increased during clinical worsening. Additionally, CA-125 showed a significant correlation with the pulmonary capillary wedge pressure (PCWP). D’Aloia et al. [17] analyzed 286 patients with chronic HF and found that higher CA-125 levels were associated with increased mortality and HF hospitalization compared to CA-125 < 35 U/mL. They also observed a strong association between CA-125 levels and PCWP and right-atrial pressure. Soler et al. [18] studied 2961 HF patients, focusing on severe tricuspid regurgitation (TR), and identified CA-125 as a superior prognostic biomarker for mortality compared to Nt-proBNP in patients with TR. Conversely, Yilmaz et al. [19] compared 40 healthy individuals with 40 patients suffering from moderate-to-severe COPD and found higher CA-125 levels in COPD patients. CA-125 also correlated significantly with the systolic pulmonary artery pressure and tricuspid annular plane systolic excursion. In addition, a recent study aimed to determine the association of CA125 with all-cause mortality at 6 months in 245 patients with STEMI undergoing coronary angioplasty; CA125 presented a similar performance of predicting mortality as NTproBNP and hs-CRP. In this study, patients with CA125 ≥ 11.48 had a higher rate of mortality (Hazard Ratio = 2.07, 95% CI 1.13–3.77, *p* = 0.017), suggesting that elevated CA125 levels might be used to identify patients with STEMI with a higher risk of 6-month death [20].

In contrast to the existing literature, our study represents the first attempt to evaluate the potential of CA-125 as a biomarker for short-term prognosis in acute symptomatic PEs. We discovered that patients who did not survive within 30 days exhibited significantly higher CA-125 levels (123.5 U/mL ± 200 vs. 39.8 ± 121; *p* = 0.01). The bivariate analysis showed a significant association for CA-125 levels > 20 U/mL with 30-day mortality and this association was independent in the multivariate analysis adjusted by age, cancer, NT-proBNP > 600 ng/mL, and PESIs (which includes heart failure as a variable). Notably, there was no association observed between CA-125 levels and treatment complications. We hypothesize that these findings may be linked to the activation of mesothelial cells in response to serous effusion, inflammation, and changes in the volume and pressure within the right heart, leading to elevated CA-125 levels in the bloodstream. These results suggest that CA-125 may serve as a potential biomarker for enhancing risk stratification in individuals with PE. In contrast, previously, our working group evaluated the role of soluble P-selectin as a potential prognostic biomarker in PE patients, showing that sP-selectin was not useful for predicting short-term mortality or major bleeding in patients with acute symptomatic PEs [21]. Therefore, by considering the feasibility, reproducibility, and cost-effectiveness of the CA-125 measurement (less than USD 5 per patient) in comparison to other biomarkers [7,12,14], our study suggests that CA-125 could be a promising and intriguing short-term biomarker for predicting adverse outcomes in acute PEs. Nonetheless, the biological mechanisms of CA-125 in non-oncological conditions remain enigmatic, requiring further research efforts to unravel its true potential in PE prognosis.

The present study is subject to several limitations. Firstly, the relatively small sample size and the low number of events limited our ability to perform a comprehensive multivariate analysis for a more thorough evaluation of this potential association. Secondly, the single-center design may restrict the generalizability of its findings to other healthcare settings and populations. Thirdly, a lack of comparable prognostic studies hampers direct comparisons with the existing literature. Despite these limitations, the study possesses several strengths. Despite its sample size, the patient cohort closely represents those encountered in routine clinical practice. Moreover, blood samples were collected at the time of PE diagnosis. Additionally, this study represents the first prospective assessment of CA-125 concerning mortality within a very specific pathology characterized by elevated morbidity and mortality rates.

## 5. Conclusions

Our study identified a significant association between elevated CA-125 levels and short-term mortality in acute symptomatic pulmonary embolism patients. Furthermore, CA-125 shows promise as a surrogate biomarker for assessing right-heart failure across various clinical scenarios. Its affordability, widespread availability, and reproducibility make it a prime candidate for further investigation and potential implementation in routine clinical practice.

## Figures and Tables

**Table 1:** Demographic, clinical, and laboratory characteristics in our population, and comparison between survivors and non-survivors at 30 days.

Variable	Total (*n* = 164)	Survivors (*n* = 147)	Non-Survivors (*n* = 17)	*p* Value
Age, years (mean, SD)	69.8 (±17)	68.6 (±17)	80.1 (±8)	<0.01
Sex male, *n* (%)	92 (56.1)	82 (55.8)	10 (58.8)	1
BMI, kg/m^2^ (mean, SD)	27.9 (±6)	28.1 (±6)	26.1 (±4)	0.24
Hypertension, *n* (%)	83 (50.6)	73 (49.7)	10 (58.8)	0.47
Diabetes, *n* (%)	22 (13.4)	18 (12.2)	4 (23.5)	0.19
Chronic Heart failure, *n* (%)	14 (8.5)	8 (5.4)	6 (35.3)	<0.01
Myocardial infarction, *n* (%)	10 (6.1)	8 (5.4)	2 (11.8)	0.3
Stroke, *n* (%)	19 (11.6)	18 (12.2)	1 (5.9)	0.44
Cancer, *n* (%)	33 (20.1)	26 (17.7)	7 (41.2)	0.02
SBP, (median, P25–P75)	128 (114–143)	129 (116–143)	120 (99–135)	0.08
SBP < 90 mmHg, *n* (%)	8 (4.9)	7 (4.8)	1 (5.9)	0.96
Heart rate, (median, P25–P75)	90 (77–108)	91 (77–108)	88 (80–98)	0.53
Heart rate > 110 bpm, *n* (%)	30 (18.3)	27 (18.4)	3 (17.6)	0.94
TAPSE, (median, P25–P75)	20 (18–23)	20 (18–23)	16 (13–19)	0.03
TAPSE < 16 mm, *n* (%)	7 (4.3)	5 (5.4)	2 (33.3)	<0.01
RV disfunction, *n* (%)	59 (35.9)	53 (39.6)	6 (50)	0.48
Central PE, *n* (%)	69 (42.1)	66 (45.5)	3 (18.8)	0.04
**Laboratory findings**				
Hemoglobin, g/dL (median, P25–P75)	13.4 (11.9–14.7)	13.4 (11.9–14.7)	12.9 (11–14.2)	0.14
Leukocytes, ×1000∙µL^−1^ (median, P25–P75)	9.8 (7.8–12)	9.5 (7.7–11.7)	10.7 (9.2–15.5)	0.02
Platelets, ×1000∙µL^−1^ (median, P25–P75)	211 (168–256)	209 (170–255)	240 (165–287)	0.28
CRP, mg/dL (median, P25–P75)	30.8 (11.6–66)	30 (11–59)	59.5 (20–151)	0.01
CRP > 49 mg/dL, *n* (%)	53 (32.3)	44 (30.8)	9 (52.9)	0.07
D-dimer, ng/mL (median, P25–P75)	2648 (1342–6000)	2650 (1342–36,307)	2427 (1321–3519)	0.15
D-dimer > 1000 ng/mL, *n* (%)	138 (84.2)	124 (87.3)	14 (87.5)	0.98
NT-proBNP ng/L (median, P25–P75)	631 (212–3038)	545 (197–2342)	3419 (800–14,389)	<0.01
NT-proBNP > 600 ng/L, *n* (%)	72 (43.9)	60 (47.2)	12 (80)	0.02
CA125, U/mL (median, P25–P75)	16.4 (10.3–30.8)	14.9 (10.3–27.5)	35.4 (20.2–108.9)	<0.01
**Outcomes**				
Mortality, n (%)	17 (10.4)	-	-	-
Major bleeding, n (%)	9 (5.5)	-	-	-

BMI: body mass index; SBP: systolic blood pressure; TAPSE: tricuspid annular plane systolic excursion; RV: right ventricle; PE: pulmonary embolism; CRP: C-reactive protein; NT-proBNP: N-terminal pro-brain natriuretic peptide; CA125: carbohydrate antigen 125.

**Table 2:** Bivariate logistic regression analysis for 30-day mortality and 30-day major bleeding.

Variables	OR	95% CI		*p* Value	OR	95% CI		*p* Value
	Mortality				Major Bleeding			
Age (years)	1.07	1.03	1.1	<0.01	1.03	0.99	1.06	0.14
Sex (male)	0.9	0.36	2.29	0.83	1.72	0.47	6.31	0.41
BMI > 30	0.24	0.05	1.11	0.07	6.59	1.28	33.82	0.02
Cancer	2.82	1.04	7.7	0.04	5.16	1.41	18.96	0.01
SBP < 90 mmHg	2.01	0.41	9.98	0.39	1	-	-	-
Heart rate > 110 bpm	0.68	0.19	2.47	0.56	0.4	0.05	3.3	0.4
RV dysfunction	0.64	0.21	1.99	0.44	0.6	0.16	2.33	0.46
TAPSE < 16 mm	7.23	1.5	39.76	0.01	1.7	0.18	15.9	0.64
Central PE	0.32	0.11	1.01	0.06	0.32	0.07	1.54	0.15
CRP > 49 mg/L	2.11	0.83	5.36	0.12	1.31	0.36	4.84	0.68
Hemoglobin < 12 g/dL	2.55	0.99	6.56	0.05	4.6	1.24	17	0.02
Platelets ×1000∙µL^−1^	1	-	-	-	3.43	0.37	31.73	0.28
D-dimer ng/mL	1.3	0.28	6	0.74	1.36	0.16	11.27	0.78
NT-proBNP > 600 ng/dL	3.58	1.11	11.43	0.03	3.63	0.73	18.1	0.12
PESIs	11.2	1.45	85.75	0.02	2.11	0.43	10.27	0.35
CA125 > 20 U/mL	6.12	1.89	19.8	<0.01	1.27	0.33	4.93	0.73

BMI: body mass index; SBP: systolic blood pressure; TAPSE: tricuspid annular plane systolic excursion; RV: right ventricle; PE: pulmonary embolism; CRP: C-reactive protein; NT-proBNP: N-terminal pro-brain natriuretic peptide; PESIs: simplified Pulmonary Embolism Severity Index; CA125: carbohydrate antigen 125. OR: odds ratio; CI: confidence interval.

**Table 3:** Multivariate logistic regression analysis for 30-day mortality and 30-day major bleeding.

Variables	OR	95% CI		*p* Value	OR	95% CI		*P* Value
	Mortality				Major Bleeding			
Age (years)	1.05	1.01	1.08	0.02	-	-	-	-
BMI > 30	-	-	-	-	9.77	1.64	58.1	0.01
Cancer	1.53	0.51	4.59	0.45	2.37	0.5	11.2	0.28
Hemoglobin < 12 g/dL	-	-	-	-	3.76	0.77	18.3	0.1
NT-proBNP > 600 ng/dL	1.87	0.43	8.18	0.4	-	-	-	-
High-risk PESIs	4.15	0.45	37.9	0.21	-	-	-	-
CA125 > 20 U/mL	4.95	1.61	15.2	<0.01	-	-	-	-

BMI: body mass index; NT-proBNP: N-terminal pro-brain natriuretic peptide; PESIs: simplified Pulmonary Embolism Severity Index; CA125: carbohydrate antigen 125. OR: odds ratio; CI: confidence interval.

## Data Availability

The data that support the findings of this study are available from the corresponding author upon a reasonable request.
